# Frequency-Dependent Selection Predicts Patterns of Radiations and Biodiversity

**DOI:** 10.1371/journal.pcbi.1000892

**Published:** 2010-08-26

**Authors:** Carlos J. Melián, David Alonso, Diego P. Vázquez, James Regetz, Stefano Allesina

**Affiliations:** 1 National Center for Ecological Analysis and Synthesis, University of California, Santa Barbara, Santa Barbara, California, United States of America; 2 Center for Ecology, Evolution and Biogeochemistry, Swiss Federal Institute of Aquatic Science and Technology, Kastanienbaum, Switzerland; 3 Community and Conservation Ecology Group, University of Groningen, Groningen, The Netherlands; 4 Instituto Argentino de Investigaciones de las Zonas Áridas, Consejo Nacional de Investigaciones Científicas y Técnicas, Mendoza, Argentina; 5 Instituto de Ciencias Básicas, Universidad Nacional de Cuyo, Centro Universitario, Mendoza, Argentina; 6 Department of Ecology and Evolution, University of Chicago, Chicago, Illinois, United States of America; University of Bern, Switzerland

## Abstract

Most empirical studies support a decline in speciation rates through time, although evidence for constant speciation rates also exists. Declining rates have been explained by invoking pre-existing niches, whereas constant rates have been attributed to non-adaptive processes such as sexual selection and mutation. Trends in speciation rate and the processes underlying it remain unclear, representing a critical information gap in understanding patterns of global diversity. Here we show that the temporal trend in the speciation rate can also be explained by frequency-dependent selection. We construct a frequency-dependent and DNA sequence-based model of speciation. We compare our model to empirical diversity patterns observed for cichlid fish and Darwin's finches, two classic systems for which speciation rates and richness data exist. Negative frequency-dependent selection predicts well both the declining speciation rate found in cichlid fish and explains their species richness. For groups like the Darwin's finches, in which speciation rates are constant and diversity is lower, speciation rate is better explained by a model without frequency-dependent selection. Our analysis shows that differences in diversity may be driven by incipient species abundance with frequency-dependent selection. Our results demonstrate that genetic-distance-based speciation and frequency-dependent selection are sufficient to explain the high diversity observed in natural systems and, importantly, predict decay through time in speciation rate in the absence of pre-existing niches.

## Author Summary

Ecological opportunity, or filling a pre-existing unoccupied adaptive zone, is considered the dominant mechanism explaining the initial explosion of diversity. Although this type of niche filling can explain rates of diversification in some lineages, it is not sufficient for a radiation to occur. Instead of attributing the propensity to have an explosion of new species to external influences like niche availability, an alternative hypothesis can be based in frequency-dependent selection driven by the ecology in which organisms are embedded or endogenous sources mediated by gametes during fertilization. We show that genome diversification driven by higher reproductive probability of rare genotypes generates rapid initial speciation followed by a plateau with very low speciation rates, as shown by most empirical data. The absence of advantage of rare genotypes generates speciation events at constant rates. We predict decline over time and constant speciation rate in the cichlids and Darwin's finches, respectively, thus providing an alternative hypothesis for the origin of radiations and biodiversity in the absence of pre-existing niche filling. In addition to predicting observed temporal trends in diversification, our analysis also highlights new mechanistic models of evolutionary biodiversity dynamics that may become suitable to generate neutral models for testing observed patterns in speciation rates and species diversity.

## Introduction

Speciation is one of the most complex phenomena in nature, yet the effects of its tempo and mode for biodiversity patterns are still controversial [1], [2]. Pre-existing niches is considered the dominant mechanism explaining the initial explosion of diversity observed in radiations [3]–[7]. In contrast, non-adaptive radiations [8], [9] driven by niche-independent mechanisms such as sexual selection, rapid range expansion across multiple barriers or the simultaneous formation of multiple geographical barriers, dispersal limitation or isolation by distance without physical barriers due to genetic incompatibilities do not predict such a temporal trend of declining speciation rates during a radiation [10]–[13].

Although ecological opportunity (the availability of an unoccupied adaptive zone) or rapid range expansion across multiple barriers can explain rates of diversification in some radiating lineages, this is not sufficient for a radiation to occur [14]–[17]. Instead of attributing the propensity to have a radiation with decaying through time speciation rates to external influences like niche availability or rapid range expansion an alternative hypothesis can be based in the genome properties evolved during the evolutionary history of organisms. We explore this hypothesis using two models, one with frequency-dependent selection and one without it. Both models involve DNA sequence-based evolution of populations via a process of sexual reproduction, assortative mating, mutation, and genetic-distance-based speciation.

The models we have analyzed in the present study are similar in spirit to previous speciation models [12], [13], [18], [19] but different in two key details: (1) no approximations of the tempo and mode of speciation incorporating sexual reproduction and frequency-dependent selection have previously been shown to explain observed patterns of decay through time of the speciation rate during a radiation without invoking pre-existing niches. Furthermore, we show that the decay through time of the speciation rate during a radiation without invoking pre-existing niches has dramatic consequences to species richness and diversity, and (2) we contrast the models with two small radiations for a broad range of parameter values: the Tilapia cichlid genus [11] and the Darwin's finches [20], two groups where assortative mating has been previously documented [21]–[24]. We note that larger radiations cannot be handled computationally. This represents a current limitation to explore broad patterns of speciation and diversity that requires further research.

**Figure 1:**
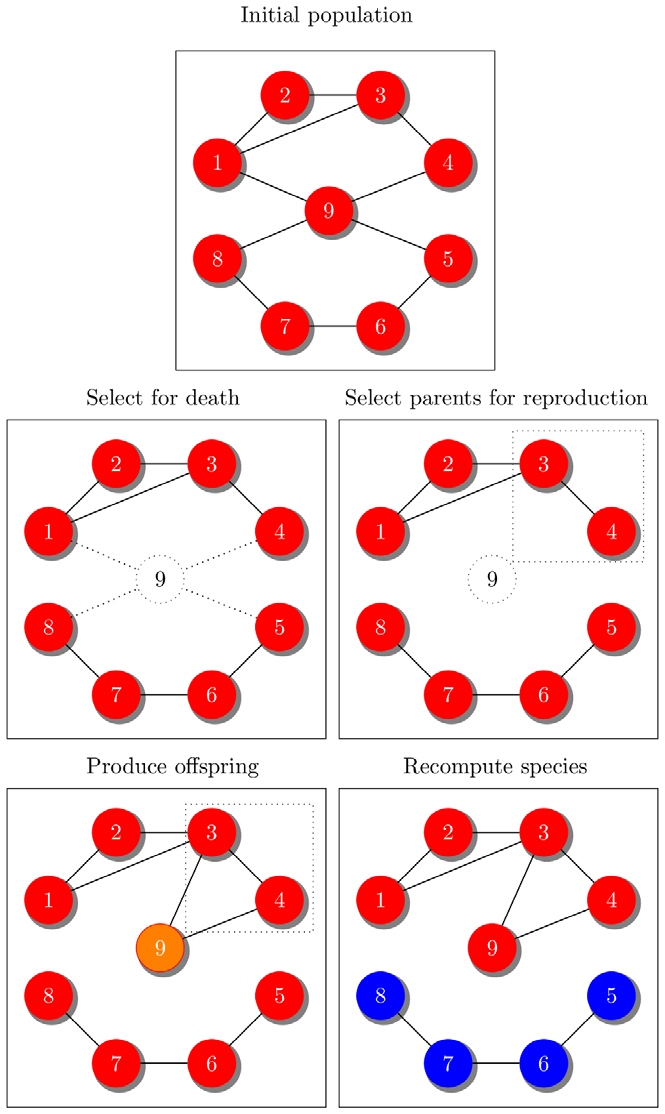
Model of evolution. In each time step, first an individual dies. Second, parents are selected for reproduction (dotted square). Third, the dead individual is replaced by an offspring. Lastly, we recompute the species number and abundance. We then repeat the cycle. In this case the graph has two species with 5 (red circles) and 4 (blue circles) individuals.

**Figure 2:**
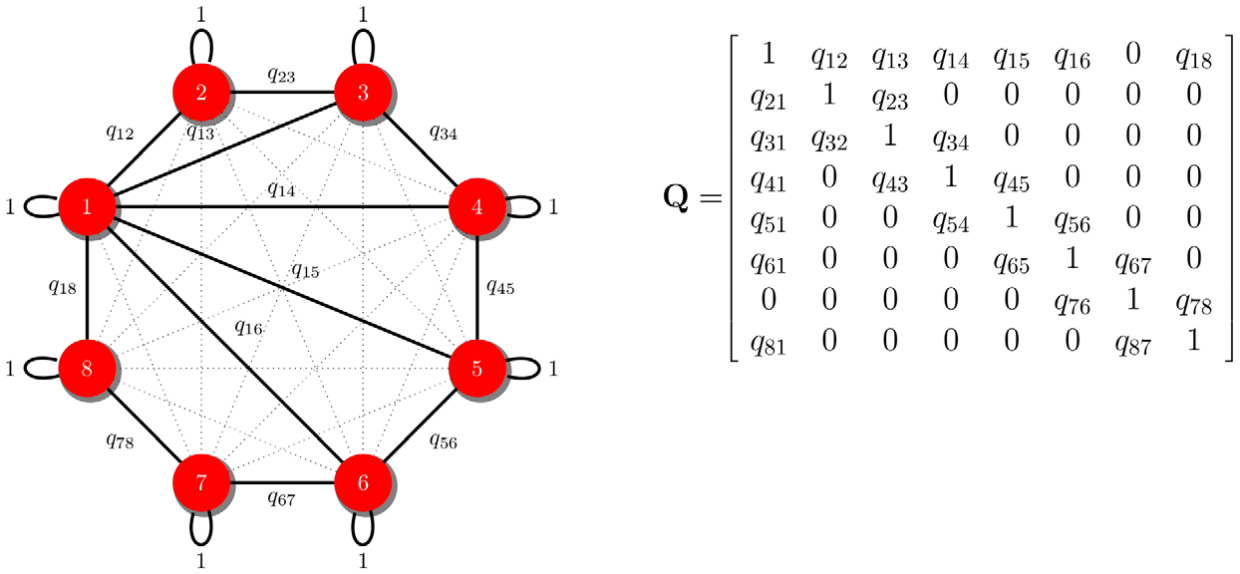
Genetic similarity matrix. In an evolutionary graph, individuals occupy the vertices of a graph. In each time step, an individual is selected with a probability proportional to its fitness. In the model without frequency-dependent selection, individuals are selected randomly. In the frequency-dependent selection model, individuals with few connections, and therefore with more rare alleles, have more success at mating and their alleles spread quickly through the population. The process is described by a symmetric genetic similarity matrix 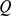, where 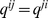 denotes the genetic similarity between individual 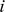 and 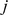. Dotted links represented by 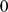 in the 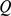 matrix denote the similarity values 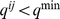, indicating reproductive incompatibility.

We simulated the evolution of a population whose members, at the beginning, have identical genomes. The population evolves under the combined influences of sexual reproduction and mutation (Text S1). During reproduction, potential mates are identified from those whose genomes are sufficiently similar to that of the reproducing individual (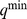). This parameter implicitly captures the effects of the accumulation of genetic incompatibilities by prezygotic or postzygotic reproductive isolation [18], [25]–[27]. A mate is chosen from this set at random. An offspring is then dispersed in the environment. This minimal form of mating called assortative mating [13], [28], [29] is sufficient for speciation at least when there is no genetic linkage [18], [19]. Genomic similarity between two individuals is defined as the proportion of identical nucleotides along the genome. The genomic similarity among individuals can be represented by an evolutionary graph in which nodes are individuals and edges connect reproductively compatible individuals [30] (Figs. 1 and 2). We identify a species as a group of organisms reproductively separated from all the others by genetic restriction on mating, but connected among themselves by the same condition. Thus, two individuals connected at least by one pathway through the evolutionary graph are considered conspecific, even if the two individuals themselves are reproductively incompatible.

We consider three main assumptions that allow us to approximate the tempo of speciation and also to identify the conditions for each of two alternative modes of speciation in the evolutionary graph: (1) Our density of individuals is one per site, and these numbers are kept constant by assuming zero-sum dynamics. Birth-death zero-sum stochastic models are equivalent to their non zero-sum counterparts at stationarity [31]; (2) Factors influencing speciation may differ between regions of the genome, and regions of the genome involved in reproductive isolation may differ between taxa and the temporal stages of the speciation process [32]. In our model, the genome of each individual is considered effectively infinite (i.e., a very large string of nucleotides, Text S1), and (3) The mate choice function explaining the viability of the offspring is given by a step-shaped function. This is the simplest representation of Dobzhansky-Muller reproductive incompatibility [33]–[35]. Functions with equal viability in a range [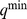, 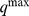] (see Material and Methods), declining linearly and exponentially [12] give qualitatively the same results as the results presented here using the step-shaped function.

At the beginning of the simulation, all individuals are reproductively compatible, corresponding to a completely connected graph. Because of mutations that can eventually reduce genetic similarity below the threshold required for mating, the graph will lose connections as generations pass (Fig. 1). The rate at which connections are lost in the evolutionary graph, and thus the tempo of speciation, depends on the mechanisms driving genome diversification.

To explore the tempo of speciation and its implications for biodiversity patterns, we generated a second model with negative frequency-dependent selection. In this model there are not external factors creating pre-existing niches, which can be populated only by individuals of a specific genotype and can be filled up to a carrying capacity. In contrast, any rare genotype has higher fitness than common types. The reason may be natural selection driven by the ecology in which the organism is embedded (e.g., bacteria or pathogens attacking reproductive proteins of common types without altering the probability to die among individuals) [36], [37] or some form of sexual selection that lead to rare-type advantage (e.g., sexual conflict, molecular incompatibility or heterozygote advantage in sexually selected genes) [38]–[40] and have more success at mating, whereas common types are likely–but not guaranteed–to become rare. Despite potential costs of the rare types (i.e., Allee effects, mating costs, etc), experimental and theoretical studies have shown that the selective value of a given genotype is often a function of its frequency in the population [41]–[46]. In summary, frequency-dependent selection in this context is a type of sexual selection with niches not imposed from outside the system but created by rare types with greater mating success that can spread their alleles more quickly through the population. Apart from the asymmetry introduced by the different reproductive probabilities at the individual level, these two models are identical (Text S1).

## Results

With appropriate parameter values satisfying the mathematical condition required for speciation (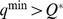 where 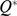 is the genetic similarity matrix at equilibrium) both models can produce speciation events (i.e., sexual isolation of subpopulations in the genome space, Equation A-30 and Box 1 in Text S1).

We identified two distinct modes of speciation that can, under the right conditions, occur in the evolving graph: mutation-induced speciation and fission (Fig. 1). Mutation-induced speciation happens when a newly produced offspring is disconnected from its parents. This form of speciation requires the mutation rate to exceed some minimum value (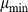) necessary to satisfy the inequality 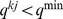, where 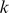 is the offspring and 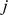 are the parents of 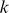 (Fig. 2). Because the minimum number of steps equals 1, the minimum mutation rate to have mutation-induced speciation is given by:
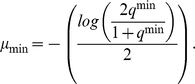
(1)For example, if offspring become inviable once genetic divergence exceeds 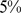 (i.e., 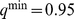), then the minimum mutation rate needed to achieve mutation-induced speciation is 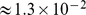. There is a second mode of speciation which is also a consequence of mutations in the evolutionary graph. We call this mode “fission” because it takes place when the death of an individual breaks a link in what was the sole genetic pathway connecting some members of a species; this gives rise to one or more new species. Because of the strict condition for mutation-induced speciation to happen, fission is the only mode of speciation in the biologically relevant portion of model parameter space (Section A3 in Text S1).

**Figure 3:**
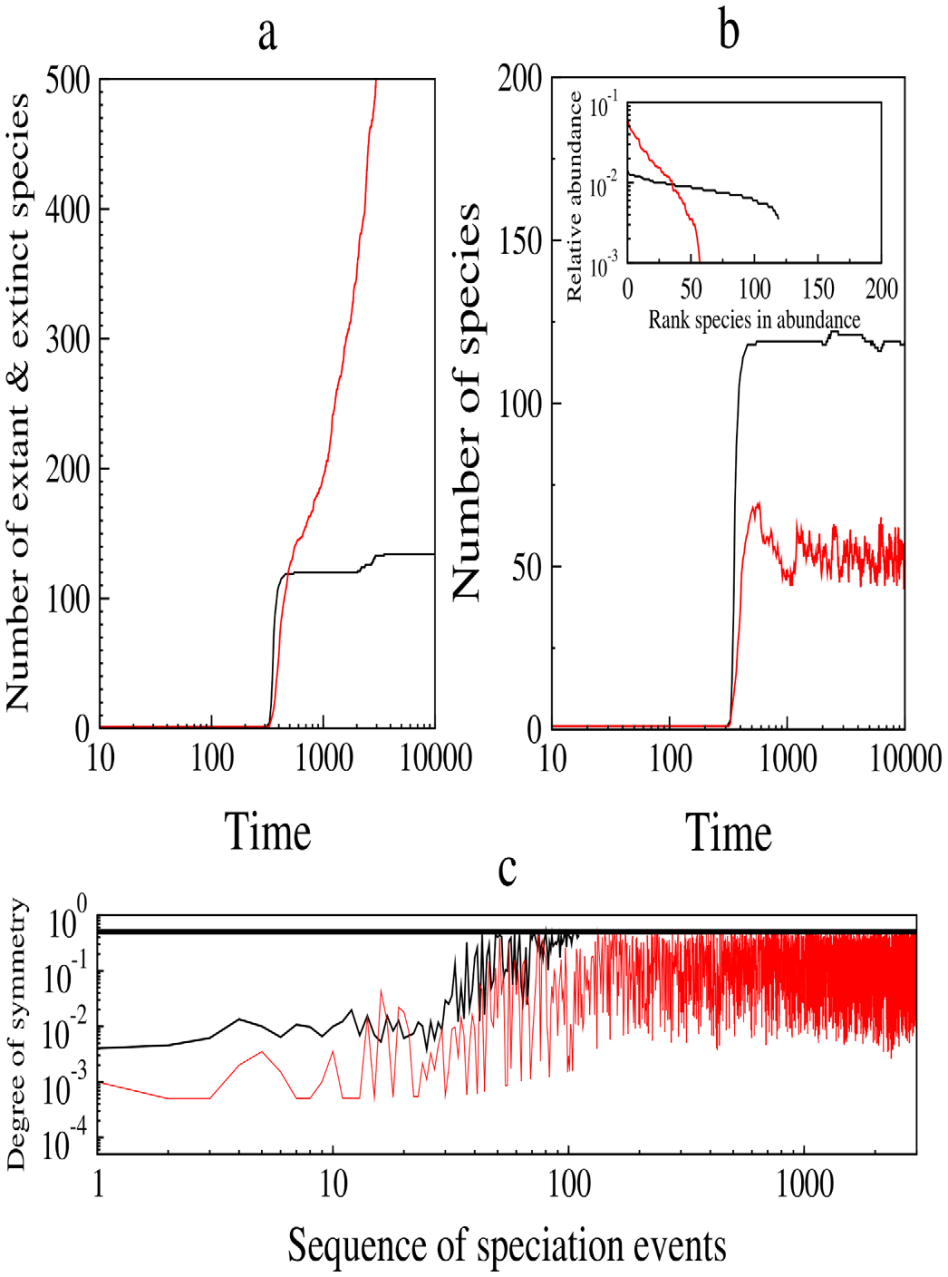
Radiations, number of species, and diversity (theory). a, Simulated total number of species (both extant and extinct) as a function of time for the model with (black, also used for b and c) and without frequency-dependent selection (red). 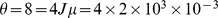, and the minimum genetic similarity value, 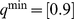, also used for b and c. Time measured in generations. After a transient phase, speciation events occur at a nearly constant rate in the model without frequency-dependent selection. In contrast, the frequency-dependent selection scenario shows a rapid series of fission speciation events followed by a plateau with very low speciation and extinctions events. b, Simulated number of extant species as a function of time for the model with and without frequency-dependent selection. Insets represent the species abundance distribution at stationarity. x and y-axis represent the rank in species abundance from the most common to the most rare and the relative abundance of each species in the community, respectively. Frequency-dependent selection produces more extant species and higher diversity (inset in b). c, Simulated abundance symmetry of the new species after each speciation event. We measured the degree of symmetry in each speciation event as 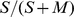, where 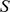 and 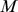 are the size of the smallest new species and the mother species, respectively. Perfect symmetry means that the new species abundance is identical to the mother species abundance; low value means the new species abundance is much smaller than that of the mother species. Thick line represents perfect symmetry.

The speciation rate (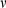) in the genetic similarity matrix (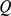) has two different dynamics according to the initial minimum genetic similarity value to have fertile offspring (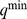):
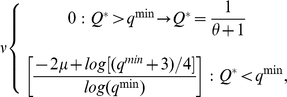
(2)where 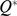 is the expected mean genetic similarity in the matrix 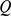 at equilibrium [18], 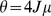 with 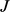 the population size. If 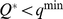, then 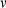 is the rate of dropping links in the evolutionary graph that is proportional to the speciation rate for the model without frequency-dependent selection (Fig. 2 and Text S1). Fitting 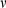 to the speciation rates obtained via simulation yielded least-squares regression coefficient estimates of 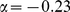 and the slope 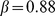 (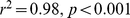):
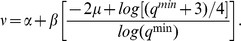
(3)This approximation suggests that the long term rate of speciation is independent of population size (Section A3 and Figs. 1 and 3 in Text S1).

**Figure 4:**
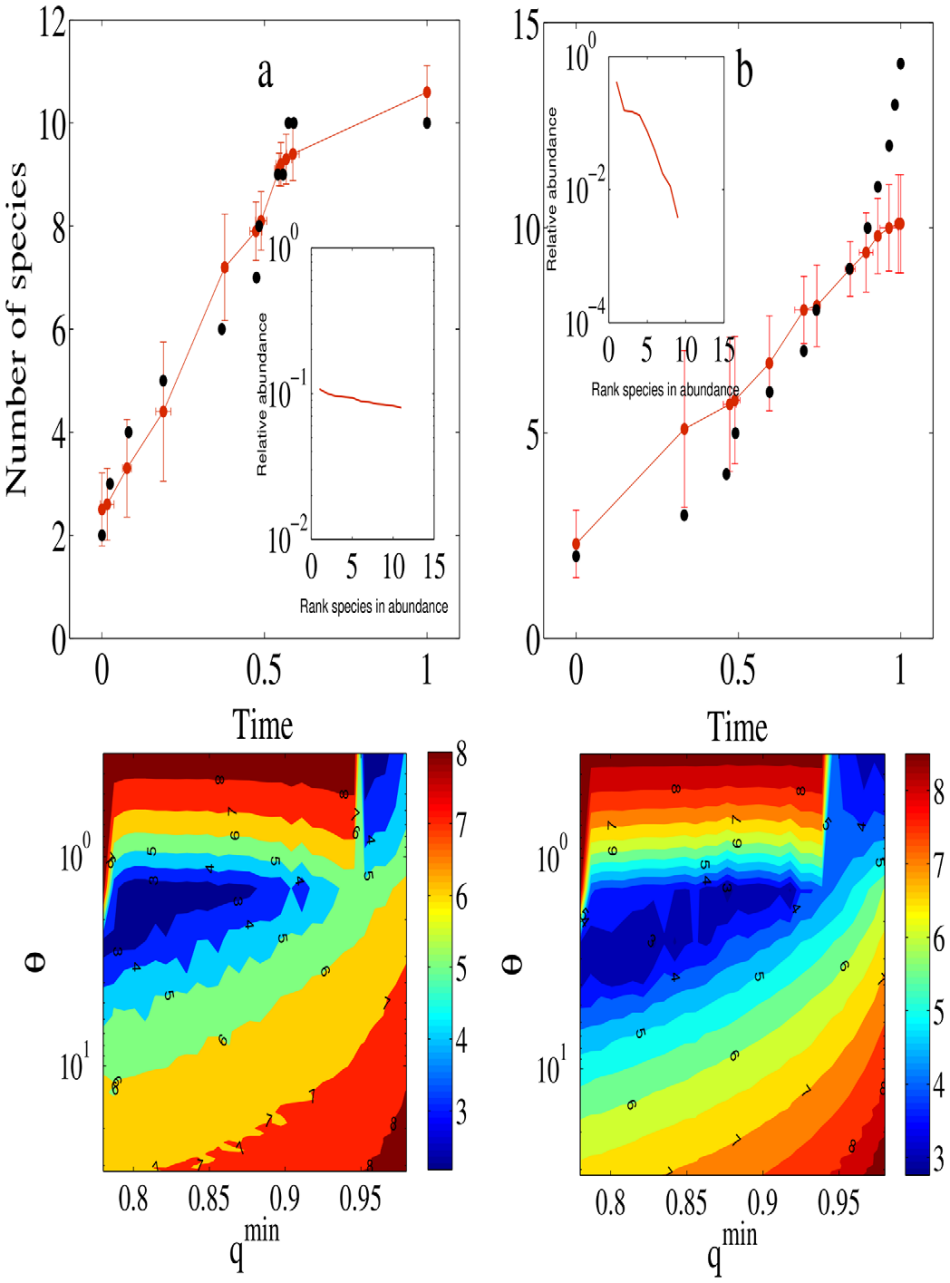
Radiations, number of species, and diversity (data). a, Empirical (black circles) and predicted (red, 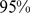 CI, Methods) species number and speciation events through time for the 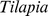 cichlid genus [61] in the Lake Barombi Mbo Lake. The best fit is given by the frequency-dependent selection model (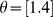, 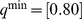 and 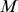 = 7.8 (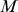 = 9.8 for the model without frequency-dependent selection, see Methods). Inset in a is the relative species abundance at stationarity given by the parameter combination that best describe the data. b, Empirical (black circles) and predicted (red, 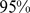 CI) species number and speciation events through time for the Darwin's finches [20]. The model without frequency-dependent selection has a slightly lower minimized value than the model with frequency-dependent selection (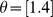, 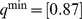 and 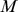 = 15.8 vs. 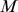 = 15.9 for the model with frequency-dependent selection). Inset in b is the relative species abundance at stationarity given by the parameter combination that best describe the data. Bottom, Parameter combinations explored for the 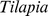 genus (left) and the Darwin's finches (right). Coloring indicates the likelihood value associated with different combinations of parameter values, with the region of “best fit” given by the dark blue area (Methods). The surface was plotted as log(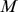) for better clarity of the isoclines. Note that 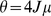 applies to a broad range of plausible empirical values of 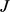 and 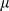.

The models generate changes over time in the tempo of speciation, the distribution of incipient species abundance, and both the number and diversity of contemporary species. In Figs. 3 and 4, we summarize the following two key predictions for the species number through time and species richness consistent with Darwin's finches and cichlid fish.

First, we predict that whether the rate of speciation will remain constant or decline over time depends on the addition of frequency-dependent selection. Fig. 3a shows how the number of extinct and extant species varies over time. After a transient period, during which mutation introduces genetic variability into the initially identical population, the number of species increases rapidly. The two models then diverge dramatically. In the model without frequency-dependent selection, speciation rate remains constant. This pattern is consistent with the literature on whole-tree cladistic analysis [47], the record of marine invertebrate fossils from the Phanerozoic eon [48], and (over shorter time frames) observed genetic differences among North American songbirds [49]. The number of contemporary species (Fig. 3b), diversity (Inset Fig. 3b), and the abundance of the new species (Fig. 3c) are lower than in the frequency-dependent model. In the frequency-dependent case, rapid speciation is followed by a plateau with few speciation events, consistent with molecular data for several groups showing declining speciation rates through time [16], [50]–[53]. This model predicts a greater number of contemporary species, higher diversity, and a more symmetric abundance distribution of incipient species; these are all attributes of rapid radiations.

Second, frequency-dependent selection reproduces cichlid radiations in absence of pre-existing niches and the absence of frequency-dependent selection generates the Darwin's finches radiation. Fig. 4a and 4b show the best fit to the data for the number of species and speciation events through time. We predict decline over time and constant speciation rate in the cichlids and Darwin's finches with and without frequency-dependent selection, respectively (data not shown). The expected distributions of species abundance derived from those predictions depart dramatically. For the 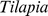 genus, the model predicts high diversity, with most species having similar abundances (inset Fig. 4a); for the Darwin's finches, the model predicts much lower species diversity, with most species being rare (insets Fig. 4b).

## Discussion

Most speciation studies have concluded that sympatric speciation only occurs if a stringent set of conditions is met [4], [7]. Likewise, for the models we have explored, sympatric speciation can be highly unlikely or even impossible in biologically relevant areas of parameter space (i.e., 
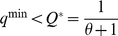
, where 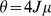, Text S1). Note, however, that even though geographical barriers and dispersal limitation, and/or range expansion have played an important role in radiations, those factors do not generate decay through time in speciation rate in the absence of niche filling [10]–[13] (see also Fig. 5 in Text S1). Interestingly, the absence of frequency-dependent selection does not capture the exponential growth in number of species in the last stage of the Darwin's finches radiation. Time lag for extinctions [50], taxonomic splitting but also the increase in heterogeneity with time in the Galápagos archipelago (i.e., more islands, habitat diversity and food types) [20] are some of the factors that may hamper model predictions in this case. Nevertheless, the balance of results for both the cichlids and the Darwin's finches suggest that neutral and frequency-dependent selection mechanisms have played a role in radiating lineages.

Current biodiversity theory, from population genetics [13] to island biogeography and its extensions [54], explain species abundance patterns for many groups, but cannot predict different trends in the tempo of speciation nor their implications for radiations and diversity patterns. The models we have explored generate alternative tempo of speciation and these models can be compared with the patterns of diversity underlying classic radiations. In the context of these models, we have also determined the conditions necessary for the mutation-induced mode of speciation; if these are not met, then fission must be the only speciation mode. Finally, we have shown that frequency-dependent selection generates more symmetric and larger incipient species abundances, resulting in lower extinction rates. These results reinforce the notion that the incipient species abundance can have a dramatic impact on contemporary diversity patterns [54], and suggest that both the tempo and mode of speciation themselves have a large effect on current community dynamics.

Alternative models of speciation that incorporate additional molecular or ecological components exist (i.e., spatial heterogeneity and dispersal limitation [19], [55], recombination rate, insertions and deletions [56] and the explicit mechanisms that cause genetic incompatibilities [57], [58]); however, it is not yet possible to evaluate those models with speciation rates and diversity data. Fitting models with a large number of parameters remains a challenge for the future - we have shown that a quasi-likelihood method offer a powerful approach.

In summary, the particular mechanisms underlying the dynamics of the evolutionary graph affect the tempo of speciation and diversity, but we nevertheless find theoretical distributions in agreement with the observed patterns of radiations and biodiversity for diverse taxa. Underlying the result are two simple models of a sexually reproducing population with and without frequency-dependent selection and with mating restrictions that depend on genetic distance. By examining these models under different parameter combinations and confronting them with data, we conclude that the properties of genomes during lineage diversification may influence patterns of radiations and biodiversity and the pre-existing environmental niches are not necessary for radiations to occur.

## Materials and Methods

Our simulation is a stochastic, individual-based, zero-sum birth and death model of a sexual population with overlapping generations and age-independent birth and death rates. For the simulations reported in the paper, we considered 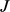 haploid and hermaphroditic individuals where only one individual can exist in each site. Genomes consist of an infinite string of nucleotides and the genomic similarity between two individuals is defined as the proportion of identical nucleotides along the genome. Reproduction starts with a randomly selected individual looking for a mate among all the sufficiently similar individuals. To qualify, an individual must have a genetic similarity greater than the minimum value required for fertile offspring. From all such potential mates, we select the second parent at random. In the frequency-dependent selection model, individuals with few connections, and therefore with more rare alleles, have more success at mating and their alleles spread quickly through the population.

Mating produces a haploid offspring that differs from both parents following free recombination and mutation (Text S1). Each nucleotide is inherited from one of the parents with the same probability. The results reported here are for asynchronous mating. Synchronous mating gave similar results, although speciation times were typically longer. According to tests of multiple model variants in the model without frequency-dependent selection, including parameter variation, self-incompatibility (i.e., by adding a 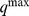 to limit the reproduction of excessively similar individuals, Fig. 5a in Text S1), and mating and dispersal limited to adjacent patches (i.e., 8-patch Moore neighborhood) with and without a wrapped torus (Fig. 5b in Text S1), our results apply quite generally, with the key required properties to generate declining through time speciation rates being the limitations on genetic distance associated with mating and the frequency-dependent selection mechanism.

Results for Fig. 3 are obtained by time-averaging over 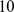 replicates lasting 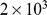 generations each. Given 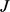 individuals in the initial population, a generation is an update of 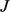 time steps. Parameter variation does not affect the overall behavior.

Results for Fig. 4 are obtained after 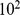 replicates for each parameter combination lasting 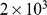 generations each. We sampled the transients (each generation) and the steady state at the end of each replicate for the species through time and species abundance. We have explored 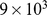 parameter combinations in the range 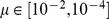, 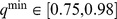, and community size, 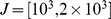 that satisfy the mathematical condition required for speciation (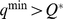, equation A-30 and Box 1 in Text S1). Our results apply quite generally in a broad range of community size (Fig. 3 in Text S1) and speciation rates (Fig. 4 in Text S1).

The fit to the number of species and speciation events through time was done following these steps: 1) Normalize time for observed data and each simulation from the first speciation event to present time within the range [0, 1], 2) From each possible interval, starting with the size of the data until the size of the output in each simulation (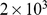 generations with increments of 1 generation at each time), we generated the sequence of speciation times that minimizes the difference with the observed speciation times, and 3) Identify the best fit as the one that minimizes the sum of the absolute values of the misfits:
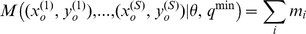
(4)where 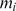 is defined as 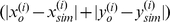, i.e., in terms of the misfit between observed and simulated species richness, 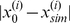, and the misfit in the timing of speciation events 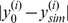. 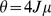 and 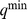 are our model parameters. Our search is performed for a broad range of plausible empirical values for 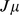 and 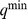 constant and satisfying 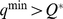 (Text S1).

If our errors per data point are a random variable 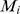 following the exponential distribution, 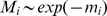, and, assuming error independence, our measure of misfit 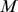 is the model negative log-likelihood [59]. Confidence intervals have been calculated by taking the percentiles 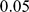 and 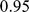 from the distributions of values of different model replicates. Model replicates were generated with the best parameter estimates for 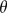 and 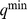 along with a family of pairs within 2 log-likelihood units away from the minimum [60] (Fig. 4 in Text S1).

## Supporting Information

Text S1 Frequency-dependent selection predicts patterns of radiations and biodiversity.
(0.22 MB PDF)
