# Supplementary material for: Frequency-Dependent Selection Predicts Patterns of Radiations and Biodiversity
Source: PLoS Comput Biol. 2010 Aug 26;6(8):e1000892. doi: 10.1371/journal.pcbi.1000892 (PMC2928887; doi:10.1371/journal.pcbi.1000892)
Supplement: Text S1 — Frequency-dependent selection predicts patterns of radiations and biodiversity. (0.22 MB PDF) [file pcbi.1000892.s001.pdf]

Text S1:
Frequency-dependent selection predicts patterns of radiations and biodiversity

Carlos J. Melián[1]*, David Alonso[2], Diego P. Vázquez[3,4] and James Regetz[1] and Stefano Allesina[5]

[1]National Center for Ecological Analysis and Synthesis,
University of California, 735 State St., Suite 300,
Santa Barbara, CA 93101, USA.
[2] Community and Conservation Ecology Group, University of Groningen
Haren, Groningen, The Netherlands.
[3]Inst. Argentino de Investigaciones de las Zonas Áridas,
CONICET, CC 507, AR-5500, Mendoza, Argentina.
[4]Instituto de Ciencias Básicas, Universidad Nacional de Cuyo,
Centro Universitario, M5502MJA Mendoza, Argentina.
[5]Department of Ecology and Evolution, University of Chicago,
1101 E 57th Street, Chicago, IL 60637, USA.

June 10, 2010

*To whom correspondence should be addressed. E-mail: melian@nceas.ucsb.edu, phone: +1-805-892-2529, fax: +1-805-892-2510.

A Text S1

We first describe in detail the way we have calculated coancestry in overlapping generations using a DNA model of evolution in haploid individuals (section A-1). This derivation has two main goals: 1) derive the expected mean genetic similarity and its relation with the condition to have speciation, and 2) derive the implementation we have used in the simulations (Box 1). Second, we compare the two models introduced in the main text with regard to reproduction (section A-2, "Reproduction dynamics"). Third, we describe the method for estimating the expected speciation rate in the model without frequency-dependent selection and the minimum mutation rate to have mutation–induced speciation (section A-3, "Speciation dynamics"). Finally, we describe the expected per capita speciation rate for several parameter combinations and community sizes (Figure 3, "Parameter variation, speciation rate and community size"), the parameter combination explored to fit the models' outputs with the data (Figure 4, " Parameter variation, model analysis and fit to the data"), and the results of variants explored (Figure 5, "Variants of the model and speciation rate").

A.1 Model of DNA sequence evolution: mutation and coancestry

Models of DNA evolution based on simple base pair substitution have a long history (i.e., the infinite sites model, (1; 2; 3)), and several variants have been proposed (4; 5). More realistic extensions of those models include deletion, insertion, duplication and rearrangements of segments bases (6). Recent models also take into account instantaneous speciation, similar to the neutral theory of biodiversity but with explicit genome evolution (specifically, an identical copy of one root genome is made, each of the two genomes gets a new successor species name, and they each evolve independently thereafter, see (6)).

Our model will be similar in spirit but different in detail. Haploid individuals reproducing

sexually are represented by a sequence of L sites representing single nucleotides, and we assume infinite sites. The nucleotides have two possible states which we represent $+1$ and -1. The genome of each individual i can be written in vector notation as $(S_1^i, S_2^i, ..., S_L^i)$, where S_u^i is the uth site of individual i. The genetic similarity between individual i and individual j is defined as:

$$q^{ij} = \frac{1}{L} \sum_{u=1}^{L} S_u^i S_u^j, \tag{A-1}$$

with $q^{ij} \in$ [-1,1]. We represent the genetic similarity values between all pairs of individuals in a community in a genetic similarity matrix, Q which has elements q^{ij}. The genetic similarity in equation (A-1) can be written in terms of the fraction of identical sites (f^{ij}):

$$q^{ij} = \frac{1}{L} \left[L f^{ij} - L(1 - f^{ij}) \right] = 2f^{ij} - 1. \tag{A-2}$$

and f^{ij} is:

$$f^{ij} = \frac{1 + q^{ij}}{2}. \tag{A-3}$$

Each nucleotide is inherited at random from one of the parents, thus ignoring linkage between neighboring sites, but with a small probability of error determined by the mutation rate, which here refers to single base substitution. Say that the individual k inherited the nucleotide in site u from one of its parents G: what is the probability that k will have exactly the same nucleotide (i.e., $+1$ or -1) as G? We assume that the probability of undergoing n mutations in site u is Poisson distributed:

$$P^k(n) = \frac{e^{-\mu} \mu^n}{n!}. \tag{A-4}$$

Each mutation switches the nucleotide (i.e., $S_u^k \rightarrow -S_u^k$). Then the probability of observing an even

number of mutations (i.e., point mutations whose cumulative effect does not change the nucleotide) at site u is:

$$P(S_u^G = S_u^k) = \sum_{i=0}^{\infty} \frac{e^{-\mu}\mu^{2i}}{(2i)!} = e^{-\mu} \sum_{i=0}^{\infty} \frac{\mu^{2i}}{(2i)!} = e^{-\mu} cosh\ \mu \tag{A-5}$$

and the probability of having an odd number of mutations (i.e., mutations whose cumulative effect changes the structure of the nucleotide is:

$$P(S_u^G = -S_u^k) = \sum_{i=0}^{\infty} \frac{e^{-\mu}\mu^{2i+1}}{(2i+1)!} = e^{-\mu} \sum_{i=0}^{\infty} \frac{\mu^{2i+1}}{(2i+1)!} = e^{-\mu} sinh\ \mu \tag{A-6}$$

Note that we can have $0, 1, 2, ..., n$ mutations in site u of the new offspring k, but because $\mu\ dt$ is small, most of the probability density is concentrated in the 0. The probabilities can be found by solving the system:

$$\begin{cases} (P(S_u^G = S_u^k)) - (P(S_u^G = -S_u^k)) = e^{-\mu} cosh\ \mu - e^{-\mu} sinh\ \mu = e^{-2\mu} \\ \\ (P(S_u^G = S_u^k)) + (P(S_u^G = -S_u^k)) = \sum_{i=0}^{\infty} \frac{e^{-\mu}\mu^i}{(i)!} = e^{\mu} e^{-\mu} = 1, \end{cases} \tag{A-7}$$

thus,

$$\begin{cases} P(S_u^G = S_u^k) = \frac{1}{2}(1 + e^{-2\mu}), \\ \\ P(S_u^G = -S_u^k) = \frac{1}{2}(1 - e^{-2\mu}). \end{cases} \tag{A-8}$$

Note that this derivation is similar to that of Peliti, Serva, Higgs and Derrida, but we consider here a two state nucleotide per site model instead of a two state allele model (7; 8; 9).

Let us consider the case of an individual k generated by two parents $G_1(k)$ and $G_2(k)$ and compare k with the two parents $G_1(j)$ and $G_2(j)$ of each individual j in the population. Which is the expected fraction of nucleotides in the offspring k shared with each individual j in the population ($E[f^{kj}]$)?

There are 4 possible combinations of the parents of k and j (i.e., $(G_1(k), G_1(j))$, $(G_1(k), G_2(j))$, $(G_2(k), G_1(j))$, $(G_2(k), G_2(j))$). For simplicity we use here the derivation for two generic parents,

G_1 and G_2. In this case, each nucleotide in site u of individuals k and j is inherited from G_1 and G_2, respectively.

We have the following probabilities: a) no mutations in site u occur in the k and j individual, thus $S_u^{G_1} = S_u^{G_2}$ ($P(a)$); b) mutations in site u occur in the k and j individual, thus $S_u^{G_1} = S_u^{G_2}$ ($P(b)$), and c) mutations in site u occur in the k and not in the j individual and viceversa, thus $S_u^{G_1} \neq S_u^{G_2}$ ($P(c)$). These probabilities can be written as:

$$\begin{cases} P(a) = \left(\frac{1}{4}(1+e^{-2\mu})\right)^2 \\ \\ P(b) = \left(\frac{1}{4}(1-e^{-2\mu})\right)^2 \\ \\ P(c) = 2\left[\left(\frac{1}{2}(1-e^{-2\mu})\right)\left(\frac{1}{2}(1+e^{-2\mu})\right)\right] = \frac{1}{2}(1-e^{-4\mu}) \end{cases} \tag{A-9}$$

This is because each pair of nucleotides $S_u^k S_u^j$ contributing to the genetic similarity of k and j comes independently and with equal probability from parents G_1 and G_2. The probability of having the same nucleotide in the site u of individual k and j is:

$$P(S_u^k = S_u^j) = \left[f^{G_1G_2}P(a) + f^{G_1G_2}P(b) + (1-f^{G_1G_2})P(c)\right], \tag{A-10}$$

and substituting the probabilities from (A-9) in (A-10) we have:

$$P(S_u^k = S_u^j) = \left[f^{G_1G_2}\left(\frac{1}{4}(1+e^{-2\mu})\right)^2 + f^{G_1G_2}\left(\frac{1}{4}(1-e^{-2\mu})\right)^2 + (1-f^{G_1G_2})\frac{1}{2}(1-e^{-4\mu})\right] \tag{A-11}$$

Substituting $f^{G_1G_2} = \frac{1+q^{G_1G_2}}{2}$, from equation (A-3) gives:

$$P(S_u^k = S_u^j) = \frac{1}{8}\left[1 + e^{-4\mu}q^{G_1G_2}\right], \tag{A-12}$$

and for all the combinations we get:

$$E[f^{kj}] = \frac{1}{8}\left[4 + e^{-4\mu}(q^{G_1(k)G_1(j)} + q^{G_1(k)G_2(j)} + q^{G_2(k)G_1(j)} + q^{G_2(k)G_2(j)})\right], \tag{A-13}$$

and substituting in equation (A-2):

$$E[q^{kj}] = 2E[f^{kj}] - 1 = 1 + \frac{e^{-4\mu}}{4}(q^{G_1(k)G_1(j)} + q^{G_1(k)G_2(j)} + q^{G_2(k)G_1(j)} + q^{G_2(k)G_2(j)}) - 1. \quad \text{(A-14)}$$

From (A-14) we then get:

$$\begin{cases} E[q^{kj}] = \frac{e^{-4\mu}}{4}(q^{G_1(k)G_1(j)} + q^{G_1(k)G_2(j)} + q^{G_2(k)G_1(j)} + q^{G_2(k)G_2(j)}), \\ E[q^{kk}] = 1, \end{cases} \quad \text{(A-15)}$$

which implies we have to keep track of the parents of all the individuals j in the population. Instead of using this equation, we implemented a faster method that yields the same result (see the Box 1 below).

We can use eq. (A-15) to calculate the expected mean similarity for the population. What is the expected genetic similarity ($\overline{q}$) between each pair of individuals in population J? Consider a large population, ignoring therefore the case in which one individual has only one parent reproducing hermaphroditically, and pick up two individuals i and j at random at time t. What is the probability that those two individuals have 2, 1, or 0 parents in common? These probabilities are given by an hypergeometric distribution:

$$P_2^{ij} = \frac{\binom{2}{2}\binom{J-2}{0}}{\binom{J}{2}} = \frac{2}{J(J-1)}, \quad \text{(A-16)}$$

$$P_1^{ij} = \frac{\binom{2}{1}\binom{J-2}{1}}{\binom{J}{2}} = \frac{4(J-2)}{J(J-1)}, \quad \text{(A-17)}$$

$$P_0^{ij} = \frac{\binom{2}{0}\binom{J-2}{2}}{\binom{J}{2}} = \frac{(J-2)(J-3)}{J(J-1)}. \quad \text{(A-18)}$$

If $\overline{f}$ and $\overline{q}$ are the expected fraction of identical nucleotides and the genetic similarity, respectively, what is the expected genetic similarity in the case of 2, 1 and 0 identical parents? By substitution, we find:

$$E[f_2^{ij}] \approx \frac{1}{2} + \frac{1}{2}\overline{f}, \quad \text{(A-19)}$$

$$E[f_1^{ij}] \approx \frac{1}{4} + \frac{3}{4}\overline{f}, \quad \text{(A-20)}$$

$$E[f_0^{ij}] \approx \overline{f}, \quad \text{(A-21)}$$

and substituting the expected values $E[f^{ij}]$ in equation (A-2) we have:

$$E[q_2^{ij}] = 2(E[f_2^{ij}]) - 1 \approx 2\left(\frac{1}{2} + \frac{1}{2}\overline{f}\right) - 1 = \frac{1+\overline{q}}{2}, \tag{A-22}$$

$$E[q_1^{ij}] = 2(E[f_1^{ij}]) - 1 \approx 2\left(\frac{1}{4} + \frac{3}{4}\overline{f}\right) - 1 = \frac{1+3\overline{q}}{4}, \tag{A-23}$$

$$E[q_0^{ij}] = 2(E[f_0^{ij}]) - 1 \approx 2\left(\overline{f}\right) - 1 = \overline{q}. \tag{A-24}$$

Define $\overline{q(t)}$ as the mean genetic similarity at time t. The expected mean similarity using equation (A-16) to (A-24) is:

$$E[q(t)] = e^{-4\mu}\left[\frac{2}{J(J-1)}\left(\frac{1+\overline{q(t)}}{2}\right) + \frac{4(J-2)}{J(J-1)}\left(\frac{3\overline{q(t)}+1}{4}\right) + \left(\frac{(J-2)(J-3)}{J(J-1)}\right)\overline{q(t)}\right], \tag{A-25}$$

where the term $e^{-4\mu}$ is due to the fact that each pair of nucleotides $S_u^k S_u^j$ contributing to the genetic similarity of i and j comes independently and with equal probability from each of the parents of i and j, respectively. To specify the equilibrium condition, let $E[q(t)] = \overline{q(t)} = \overline{q} = Q^*$. Then $\overline{q}$ at steady state is:

$$\overline{q} = e^{-4\mu}\left[\frac{1+\overline{q}}{J(J-1)} + \frac{(J-2)}{J(J-1)}\left(\frac{3\overline{q}+1}{4}\right) + \left(\frac{(J-2)(J-3)}{J(J-1)}\right)\overline{q}\right], \tag{A-26}$$

and simplifying we have:

$$\overline{q} = \frac{e^{-4\mu}}{J(J-1)}\left[1 + \overline{q} + (J-2)(1+3\overline{q}) + (J-2)(J-3)\overline{q}\right], \tag{A-27}$$

and

$$\overline{q} = \frac{e^{-4\mu}}{J}\left[1 + \overline{q}(J-1)\right], \tag{A-28}$$

which gives

$$\overline{q} = \frac{1}{J(e^{4\mu}-1)+1} \tag{A-29}$$

Considering the first order Taylor expansion $e^{4\mu} = 1 + 4\mu$, equation (A-29) becomes:

$$\overline{q} = Q^* = \frac{1}{J(1 + 4\mu - 1) + 1} = \frac{1}{\theta + 1}, \quad \text{(A-30)}$$

where $\theta = 4J\mu$. The condition to have speciation is given by $q^{\min} > Q^*$ (7; 8; 9). All the parameter combinations explored in this study met this condition.

Box 1:
Using the same derivation from equation (A-9) to (A-15) we have calculated the similarity values between the parents of the offspring k (i.e., $G_1(k)$ and $G_2(k)$) and each individual j in the population. Which is the expected fraction of nucleotides in the offspring k shared with each individual j in the population ($E[f^{kj}]$)? Starting from equation (A-8), this expected fraction is:

$$\begin{aligned} E[f^{kj}] = & \frac{1}{2}\left[f^{G_1(k)j}(P(S_u^{G_1(k)} = S_u^k)) + (1 - f^{G_1(k)j})(P(S_u^{G_1(k)} = -S_u^k))\right] \\ & + \frac{1}{2}\left[f^{G_2(k)j}(P(S_u^{G_2(k)} = S_u^k)) + (1 - f^{G_2(k)j})(P(S_u^{G_2(k)} = -S_u^k))\right] \end{aligned} \quad \text{(A-31)}$$

$$\begin{aligned} E[f^{kj}] = & \frac{1}{2}\left[f^{G_1(k)j}\frac{1}{2}(1 + e^{-2\mu}) + (1 - f^{G_1(k)j})\frac{1}{2}(1 - e^{-2\mu})\right] \\ & + \frac{1}{2}\left[f^{G_2(k)j}\frac{1}{2}(1 + e^{-2\mu}) + (1 - f^{G_2(k)j})\frac{1}{2}(1 - e^{-2\mu})\right]. \end{aligned} \quad \text{(A-32)}$$

Substituting $f^{G_1(k)j} = \frac{1+q^{G_1(k)j}}{2}$ and $f^{G_2(k)j} = \frac{1+q^{G_2(k)j}}{2}$ from equation (A-3) gives:

$$\begin{aligned} E[f^{kj}] = & \frac{1}{4}\left[\frac{1+q^{G_1(k)j}}{2} + e^{-2\mu}\left(\frac{1+q^{G_1(k)j}}{2}\right) + \frac{1-q^{G_1(k)j}}{2} - e^{-2\mu}\left(\frac{1-q^{G_1(k)j}}{2}\right)\right] \\ & + \frac{1}{4}\left[\frac{1+q^{G_2(k)j}}{2} + e^{-2\mu}\left(\frac{1+q^{G_2(k)j}}{2}\right) + \frac{1-q^{G_2(k)j}}{2} - e^{-2\mu}\left(\frac{1-q^{G_2(k)j}}{2}\right)\right], \end{aligned} \quad \text{(A-33)}$$

and after simplification we obtain:

$$E[f^{kj}] = \frac{1}{4}\left[1 + e^{-2\mu}q^{G_1(k)j}\right] + \frac{1}{4}\left[1 + e^{-2\mu}q^{G_2(k)j}\right], \quad \text{(A-34)}$$

$$E[f^{kj}] = \frac{1}{4}\left[2 + e^{-2\mu}q^{G_1(k)j} + e^{-2\mu}q^{G_2(k)j}\right], \quad \text{(A-35)}$$

and substituting in equation (A-2):

$$E[q^{kj}] = 2E[f^{kj}] - 1 = 1 + \frac{e^{-2\mu}}{2}q^{G_1(k)j} + \frac{e^{-2\mu}}{2}q^{G_2(k)j} - 1. \quad \text{(A-36)}$$

From (A-36) we then get:

$$\begin{cases} E[q^{kj}] = \frac{e^{-2\mu}}{2}(q^{G_1(k)j} + q^{G_2(k)j}), \\ E[q^{kk}] = 1, \end{cases} \quad \text{(A-37)}$$

and the expected mean similarity at equilibrium is $Q^* = \frac{1}{\theta+1}$, where $\theta = 4J\mu$. Note that this is the same expected value than the previous derivation from equations (A-15) to (A-30). The implementation of this derivation is faster because we do not have to keep track of the parents of each individual in the population.

A.2 Reproduction dynamics

A.2.1 Frequency-dependent selection model

We have explained the model without frequency-dependent selection in the main text. We here described the frequency-dependent selection model. The essential difference between the model without and with frequency-dependent selection is that in the former all individuals are chosen for reproduction with the same probability, while in the latter rare genotypes have increased reproductive success.

In this model, the fitness of each individual i within each species k is inversely proportional to the total number of individuals j satisfying $q^{ij} > q^{\min}$, i. e., the total number of individuals each individual i can mate with. Thus, reproductive probability of individual i within each species decreases with the number of links or the number of genetically related mating partners.

The genetic level, using the genetic similarity among individuals, determines the speed of speciation rate and the genetic–species diversity. Each individual i of species k is chosen for reproduction according to:

$$P_{i,k} = \mathcal{N} F_{i,k}, \tag{A-38}$$

where individual fitness is defined as:

$$F_{i,k} = \frac{1}{\sum_{j=1}^{N_k} H(q^{ij} - q^{min})} \tag{A-39}$$

Thus we write:

$$P_{i,k} = \mathcal{N} \frac{1}{\sum_{j=1}^{N_k} H(q^{ij} - q^{min})} \tag{A-40}$$

where $\mathcal{N}$ is a normalization factor, N_k is the abundance of species k, and $H(\alpha)$ is

$$H(\alpha) = \begin{cases} 1 & \text{if } \alpha > 0 \\ 0 & \text{otherwise} \end{cases}$$

We now calculate the normalization factor by using the normalization requirement, i.e., by summing $P_{i,k}$ across all individuals and species 1 must be obtained:

$$\mathcal{N}\sum_{i=1}^{S}\sum_{j=1}^{N_k} F_{i,k} = 1, \tag{A-41}$$

where S is the number of species, then:

$$\mathcal{N} = \frac{1}{\sum_{i=1}^{S}\sum_{j=1}^{N_k} F_{i,k}} \tag{A-42}$$

Therefore, the probability of birth for each i individual is:

$$P_{i,k} = \frac{F_{i,k}}{\sum_{i=1}^{S}\sum_{j=1}^{N_k} F_{i,k}} \tag{A-43}$$

This model shares some ingredients with the model without frequency-dependent selection: (1) individuals have the same probability $1/J$ to be chosen for death, and (2) individuals that have the same number of potential partners have equal fitness, i.e, belonging to a given species does not confer per se fitness advantage. The model has the following additions: (1) fitness is inversely proportional to the number of genetically related mating partners; (2) individuals with rare sequences have higher probabilities of reproduction, and (3) we select the least connected parents with higher probability which implies that the offspring can inherit their low connectance, thus increasing their reproductive probability. In sum, model dynamic evolution selects for low connected individuals.

A.3 Speciation dynamics

A basic point in our models is that a single population gives rise to a whole community through two modes of speciation. Our main goal here is to describe in further detail the two speciation modes. Notice that the two models introduced in the main text do not differ in the dynamics of speciation: they only differ in the way parents are chosen for reproduction. Speciation dynamics is controlled by two input parameters: the mutation rate (μ) and the minimum genetic similarity value (q^{min}).

Model birth-death dynamics can generate two speciation modes: fission and mutation–induced speciation. Fission happens after the death of an individual. Mutation–induced speciation happens because the offspring can not mate with any individual in its previous population.

A.3.1 The expected speciation rate in the model without frequency-dependent selection

For clarity, we first derive the case with one parent. At steady state, let us assume that we have just one individual reproducing itself in a sequence (i.e., individual A_1 is the offspring of A, thus $A \rightarrow A_1 \rightarrow A_2$,..., A_n, see Fig. 1), what is the number of steps (n) at which $q^{\min} > q^{AA_n}$? From equation (A-37) we can represent the first step from A to A_1 as:

$$q^{AA_1} = x_1 = e^{-2\mu}, \tag{A-44}$$

and the second from A to A_2 as:

$$q^{AA_2} = x_2 = e^{(-2\mu)2}, \tag{A-45}$$

thus

$$q^{AA_n} = x_n = e^{(-2\mu)n}, \tag{A-46}$$

then

$$q^{\min} > e^{(-2\mu)n}, \tag{A-47}$$

and applying logarithms we obtain:

$$n = -\frac{log(q^{\min})}{2\mu}, \tag{A-48}$$

which is the number of steps to drop the link between A and A_n (i.e., dashed line in Fig. 1). The rate of dropping links, proportional to the speciation rate is:

$$\frac{1}{n} = -\frac{2\mu}{log(q^{\min})}. \tag{A-49}$$

The case for two parents is as follows. Let us assume that we have, as in the one parent case, a sequence of offspring $B \rightarrow B_1 \rightarrow B_2$,..., B_n, and that each one of these individuals mate with a given individual $C = C_1 = C_2 = C_n$ (see Fig. 2). We ask the same question as before; what is the number of steps (n) at which $q^{\min} > q^{BB_n}$? From equation (A-37) we now write the first step from B to B_1 as:

$$q^{BB_1} = x_1 = e^{-2\mu}\frac{(q^{BB} + q^{BC})}{2}, \tag{A-50}$$

where the $q^{BB} = 1$ and q^{BC} is the expected genetic similarity between parent B and parent C. We know this value is in the range [$q^{\min}$,1], thus the expected similarity value between parent B and C is $E[BC] = \omega = (1 + q^{\min})/2$. The equation then becomes:

$$q^{BB_1} = x_1 = e^{-2\mu}\frac{(1+\omega)}{2}, \tag{A-51}$$

and the second step from B to B_2 can be written as:

$$q^{BB_2} = x_2 = e^{(-2\mu)2}\left(\frac{(1+\omega)}{2}\right)^2, \tag{A-52}$$

thus

$$q^{BB_n} = x_n = e^{(-2\mu)n}\left(\frac{(1+\omega)}{2}\right)^n, \tag{A-53}$$

then

$$q^{\min} > e^{(-2\mu)n}\left(\frac{(1+\omega)}{2}\right)^n, \tag{A-54}$$

and applying logarithms result in:

$$n = \frac{log(q^{\min})}{-2\mu + log[(q^{min}+3)/4]}, \tag{A-55}$$

which is the number of steps to drop the link between B and B_n (i.e., dashed line in Fig. 2). The rate of dropping links that is proportional to the speciation rate is:

$$\frac{1}{n} = \frac{-2\mu + log[(q^{min}+3)/4]}{log(q^{\min})}. \tag{A-56}$$

This equation is a rate at which links are dropped in the evolutionary graph. It suggests that the true speciation rate should depend on this quantity. As an approximation, we studied a simple linear dependency (equation 3 in the main text) and found good agreement across different community sizes (Fig. 3).

Finally, using this approximation we can ask what is the minimum mutation rate ($\mu_{\min}$) for the mutation induced speciation mode to happen (i.e., $q^{ki} < q^{\min}$)? From equation (A-37) we have:

$$q^{\min} > \frac{e^{-2\mu}}{2}(q^{G_1(k)i} + q^{G_2(k)i}). \tag{A-57}$$

As in the two parent approximation, if we assume that the first parent (G_1) is equal to i and the expected genetic similarity between G_2 and i is equal to $q^{\min}$, then the equation becomes:

$$q^{\min} = e^{-2\mu_{\min}}\frac{(1+q^{\min})}{2}, \tag{A-58}$$

and applying logarithms result in:

$$log(q^{\min}) = -2\mu_{\min} + log\left(\frac{1+q^{\min}}{2}\right), \tag{A-59}$$

and the minimum mutation rate ($\mu_{\min}$) to have mutation–induced speciation is

$$\mu_{\min} = -\left(\frac{log\left(\frac{2q^{\min}}{1+q^{\min}}\right)}{2}\right), \tag{A-60}$$

which is the equation 1 in the main text.

B Text S1 figure legends

• **Figure 1 — Approximation speciation rate in the model without frequency-dependent selection (one parent model)**. We assume individual A reproducing itself in the sequence $A \rightarrow A_1 \rightarrow A_2$,..., A_n, where A_1 is the offspring of A, A_2 is the offspring of A_1 and so on. We can estimate the speciation rate by approximating the number of steps at which the link (x_n) between A and A_n drops (dashed line).

• **Figure 2 — Approximation speciation rate in the model without frequency-dependent selection (two parent model)**. As in the figure 1, we assume individual B reproducing in the sequence $B \rightarrow B_1 \rightarrow B_2$,..., B_n, where B_1 is the offspring of B, B_2 is the offspring of B_1, and B_{n+1} is the offspring of B_n, and that each one of these individuals mate with a given individual $C = C_1 = C_2 = C_n$. Similar to the one parent model, we can estimate the speciation rate by approximating the number of steps at which the link (x_n) between B and B_n drops.

• **Figure 3 — Parameter variation, speciation rate and community size**. Simulated (orange, black, red circles represent the mean after 10^2 replicates with 2×10^3 generations each for $J = 5 \times 10^2$, 10^3 and 2×10^3 individuals, respectively) vs expected (continuous line from eq. 2 in the main ms.) values for the per capita speciation rate. Parameter combination explored are: mutation rate (μ) = [10^{-4}, 2.5×10^{-4}, 5×10^{-4}, 7.5×10^{-4}, 10^{-3}, 2.5×10^{-3}, 5×10^{-3}, 7.5×10^{-3}, 10^{-2}], and genetic similarity values ($q^{\min}$) = [0.85, 0.90, 0.95]. The expected values were obtained using the outputs from the simulations with population size $J = 10^3$ and 2×10^3 individuals. Fitting this expression to the speciation rates obtained via simulation yielded least-squares regression coefficient estimates of $\alpha = -0.23$ and the slope $\beta = 0.88$ ($r^2 = 0.98$, $p < 0.001$, black line, equation 3 in the main ms.). In the context of these models, speciation rate is independent of population size.

• **Figure 4 — Parameter variation, model analysis and fit to the data**. **a**, Effect of mutation rate (μ) and the minimum genetic similarity value ($q^{\min}$) on the expected speciation

rate using the approximation from eq. A-56. Isoclines show parameter combinations with equal speciation rates. As expected, increasing μ and $q^{\min}$ narrow the space to have equal rates.

• **Figure 5 — Variants of the model and speciation rate**. **a**, Simulated number of extant and extinct species as a function of time for the model without frequency-dependent selection using a maximum similarity value to have fertile offspring (i.e., self-incompatibility among sufficiently similar individuals, $q^{\max} = 0.95$). Number of individuals, $J = [2 \times 10^3]$, mutation rate, $\mu = [10^{-4}]$, and the minimum similarity value, $q^{\min} = [0.9]$. Time measured in generations. As in the model without $q^{\max}$ speciation events are constant. Lines represent the replicates with the lowest (dashed line) and the highest number of speciation events after 10^2 replicates with 2×10^3 generations each. **b**, Simulated number of extant and extinct species as a function of time for the model without frequency-dependent selection using local dispersal and mating (i.e., surrounding frame of 8 neighbors). Simulations were done in a grid of 50×50 cells (1 individual per cell), with mutation rate, $\mu = [10^{-4}]$, and the minimum genetic similarity value, $q^{\min} = [0.9]$. Time measured in generations. Lines represent the replicates with the lowest (dashed line) and the highest number of speciation events after after 10^2 replicates with 2×10^3 generations each. Trends in speciation rates remain qualitatively similar to the model without frequency-dependent selection explored in the main ms. where mating and dispersal is global. Parameter variation does not affect the overall behavior.

C Supplementary figures

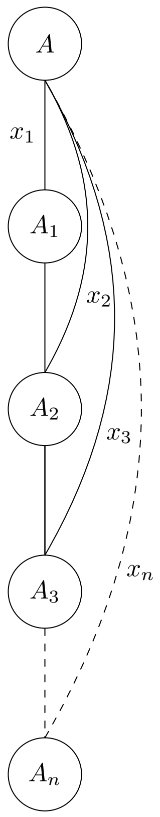

Figure 1

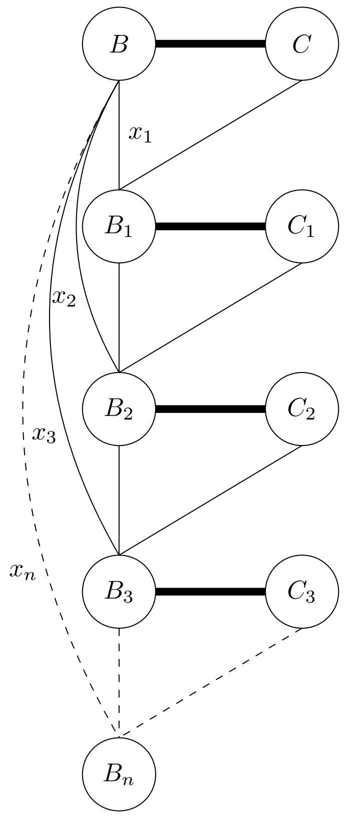

Figure 2

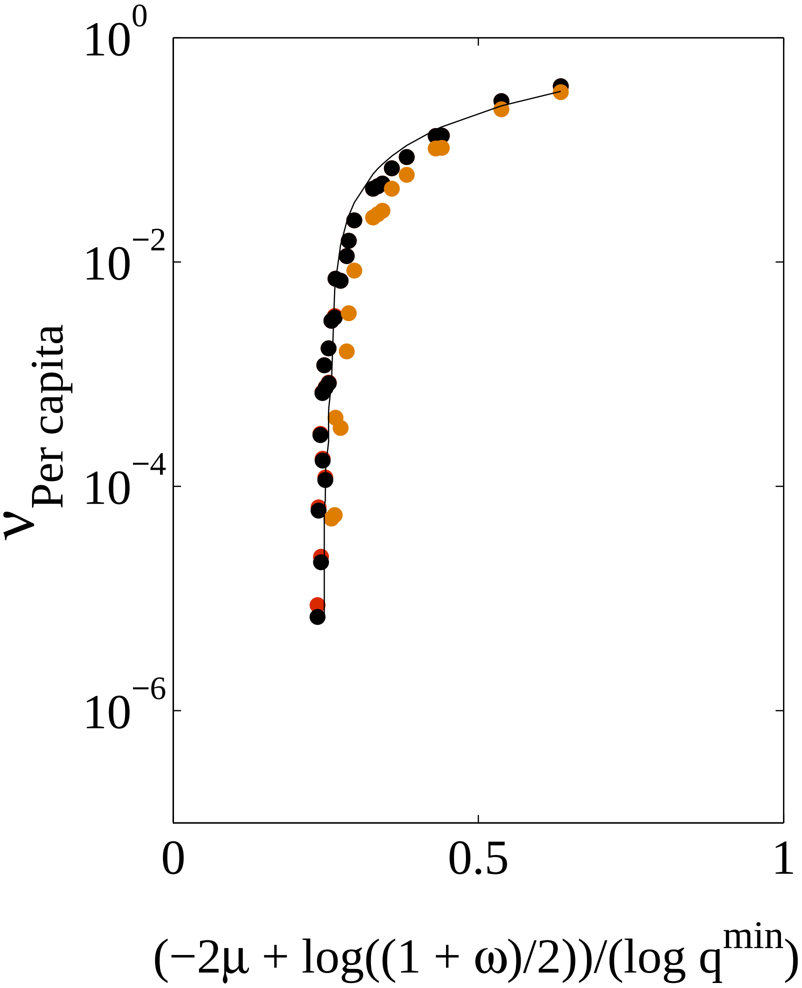

Figure 3

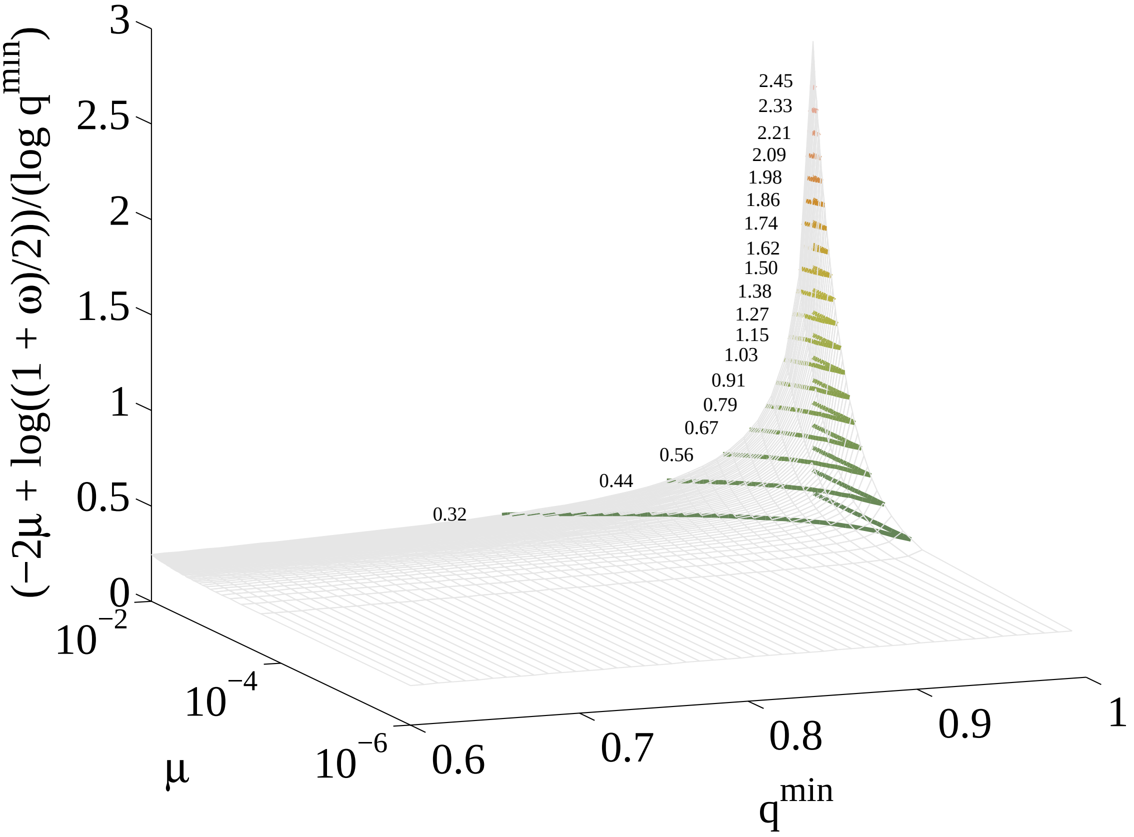
(−2μ + log((1 + ω)/2))/(log q^min)
3
2.5
2
1.5
1
0.5
0
2.45
2.33
2.21
2.09
1.98
1.86
1.74
1.62
1.50
1.38
1.27
1.15
1.03
0.91
0.79
0.67
0.56
0.44
0.32
10^−2
10^−4
10^−6
μ
0.6
0.7
0.8
0.9
1
q^min

Figure 4

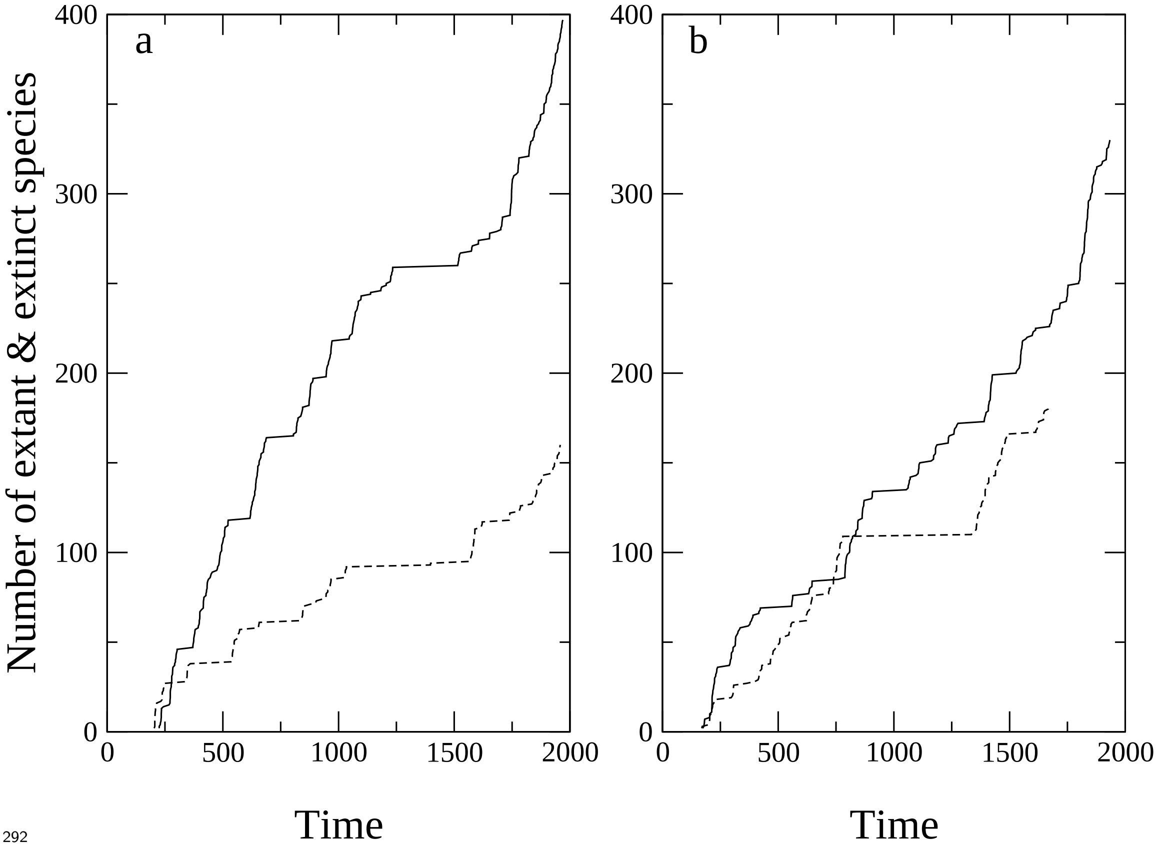
a
b
Number of extant & extinct species
Time
Time
0
100
200
300
400
0
500
1000
1500
2000

Figure 5

References

[1] Jukes TH, Cantor CR (1969) Evolution of protein molecules. In Mammalian Protein Metabolism, H. N. Munro, ed.,. (Academic Press, New York, NY, pp. 21-132).

[2] Kimura M (1980) A simple method for estimating evolutionary rates of base substitutions through comparative studies of nucleotide sequences. Journal of Molecular Evolution 16: 111-120.

[3] Kimura M (1983) The neutral theory of molecular evolution. (Cambridge University Press, Cambridge).

[4] Hasegawa M, Kishino H, Yano T (1985) Dating of human-ape splitting by a molecular clock of mitochondrial dna. Journal of Molecular Evolution 22: 160-174.

[5] Durrett R (2008) Probability models for DNA sequence evolution. (Springer, New York.).

[6] Ma J, Ratan A, Raney BJ, Suh BB, Miller W, et al. (2008) The infinite sites model of genome evolution. Proceedings of the National Academy of Sciences of the USA 105: 14254-14261.

[7] Derrida B, Peliti L (1991) Evolution in a flat fitness landscape. Bulletin of Mathematical Biology 53: 355-382.

[8] Serva M, Peliti L (1991) A statistical model of an evolving population with sexual reproduction. J Phys, A: Math Gen 24: L705-L709.

[9] Higgs PG, Derrida B (1992) Genetic distance and species formation in evolving populations. Journal of Molecular Evolution 35: 454-465.
